# On the Conformational Dynamics of β-Amyloid Forming Peptides: A Computational Perspective

**DOI:** 10.3389/fbioe.2020.00532

**Published:** 2020-06-03

**Authors:** Konda Mani Saravanan, Haiping Zhang, Huiling Zhang, Wenhui Xi, Yanjie Wei

**Affiliations:** Center for High Performance Computing, Joint Engineering Research Center for Health Big Data Intelligent Analysis Technology, Shenzhen Institutes of Advanced Technology, Chinese Academy of Sciences, Shenzhen, China

**Keywords:** amyloid, neural disorders, molecular dynamics, machine learning, conformation transition

## Abstract

Understanding the conformational dynamics of proteins and peptides involved in important functions is still a difficult task in computational structural biology. Because such conformational transitions in β-amyloid (Aβ) forming peptides play a crucial role in many neurological disorders, researchers from different scientific fields have been trying to address issues related to the folding of Aβ forming peptides together. Many theoretical models have been proposed in the recent years for studying Aβ peptides using mathematical, physicochemical, and molecular dynamics simulation, and machine learning approaches. In this article, we have comprehensively reviewed the developmental advances in the theoretical models for Aβ peptide folding and interactions, particularly in the context of neurological disorders. Furthermore, we have extensively reviewed the advances in molecular dynamics simulation as a tool used for studying the conversions between polymorphic amyloid forms and applications of using machine learning approaches in predicting Aβ peptides and aggregation-prone regions in proteins. We have also provided details on the theoretical advances in the study of Aβ peptides, which would enhance our understanding of these peptides at the molecular level and eventually lead to the development of targeted therapies for certain acute neurological disorders such as Alzheimer’s disease in the future.

## Introduction

**FIGURE 1:**
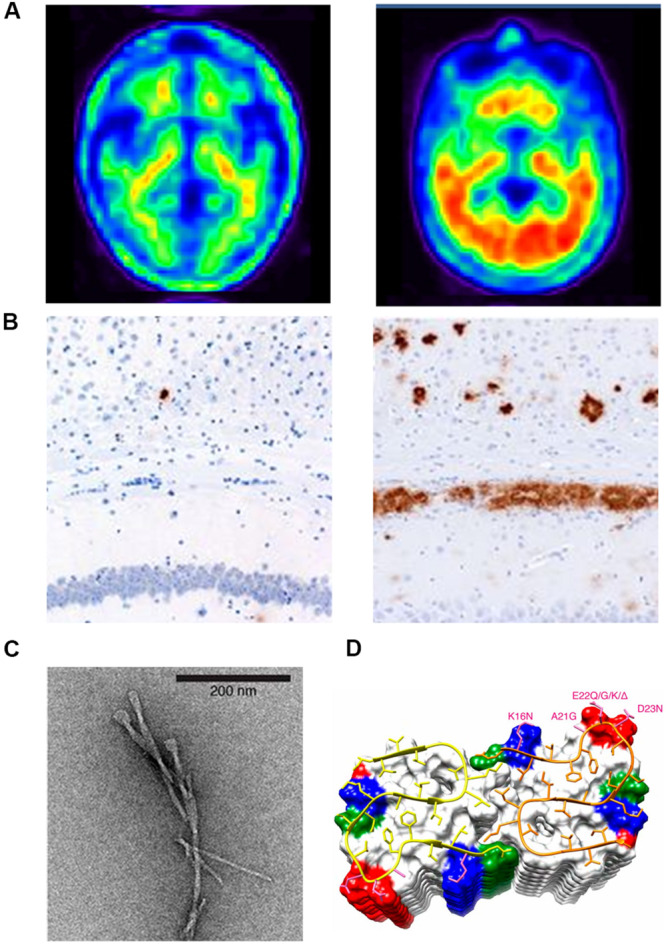
**(A)** Positron emission tomography (PET) brain images of a chronic AD patient (left) and a normal brain (right); **(B)** Aβ immunostaining of the homogenates of the hippocampus and overlying cortex obtained from aged non-transgenic mice (left) and transgenic (APP23:Gfap-luc) mice inoculated with purified transgenic (APP23) Aβ fibrils (right). **(C)** Negatively stained TEM of unstained, freeze-dried Aβ_1__–__42_ fibrils. **(D)** The microscopic structure of Aβ_15__–__42_ fibrils is shown in the ribbon diagram.

Proteins are dynamic macromolecules, it is believed that the amino acid sequence of proteins determines their tertiary structure, which is eventually responsible for its function in the cell (Anfinsen, 1973; Kabsch and Sander, 1984; Dill and MacCallum, 2012). Secondary structural elements like α-helices and β-sheets are the building blocks for the tertiary structure (Jones, 1999) and the formation of these structural elements is dictated by a combination of local and non-local interactions (Saravanan et al., 2017). Proteins are stabilized by different biophysical forces. The formation of secondary structures present in them can be altered by different factors (Minor and Kim, 1996). Several analytical methods for the identification and synthesis of flexible sequence fragments have been reported in the literature (Jacoboni et al., 2000; Yoon and Jung, 2006; Teilum et al., 2011). Proteins do not function as isolated entities; they can interact with other proteins/DNA/small molecules in the cell to form large macromolecular assemblies with distinct biological functions (Saranya et al., 2016). During the formation of these macromolecular assemblies, proteins undergo changes in their physicochemical properties and secondary structural elements, which serve as a proofreading mechanism, imparting specificity and selectivity for the binding partners (Savir and Tiusty, 2007). Protein scientists have shown that both α-helices and β-sheets can be formed with similar sequence context; such sequences in proteins are termed as “chameleon regions.” These flexible fragments in proteins can alter their structure depending on temperature, pH, phosphorylation, and other environmental factors. These conformation changes are shown to be responsible for more than 70 human diseases associated with fibril formation, the most common conformational diseases being amyloidoses, like Alzheimer’s disease (AD) and Parkinson’s disease (PD) (Egorov et al., 2015). Many of these diseases are neurological disorders, occurring because of conformational changes/misfolding/aggregation of proteins in the brain (Knowles et al., 2014). As an example, Figure 1A shows a positron emission tomography (PET) brain image of a severely affected AD’s patient (left) and a normal brain (right) (Joshi et al., 2012). The word “amyloid” is of Latin and Greek origin, and means “starch”; it was introduced in 1854 by the German scientist Rudolph Virchow, who first observed the unusual and abnormal appearance of substances during microscopic observations of the cerebrum (Bagot and Arya, 2008). Figure 1B shows an example of such kind of substances, which are usually termed as amyloid senile plaques (Stöhr et al., 2012).

X-ray crystallography, nuclear magnetic resonance (NMR) spectroscopy, and electron microscopy are three powerful techniques used for elucidating the conformation of proteins/peptides to a near-atomic resolution. NMR spectroscopy is the most efficient technique used for solving structures of small proteins/peptides present in solution. Hence, a structural model for amyloid fibrils formed by β-amyloid (Aβ) peptide is presented by solid-state NMR spectroscopy. The model is further improved by electron microscopy by adding constraints of cross-β structural motifs (Petkova et al., 2006). High-resolution magnetic resonance images (MRI) of amyloid fibrils in the cerebrum of perivascular space from different stages of disease samples are taken recently (Wälti et al., 2016; Banerjee et al., 2017). Amyloid is an extracellular protein that is characterized by a conformational transition into a β-sheet rich filament (Nelson et al., 2005). An example of such a typical filament is shown in Figure 1C, which is a high-resolution image of the two-layer Aβ (1–42) fibril model developed with solid-state NMR (Wälti et al., 2016). This detailed structure revealed that Aβ peptides form inter-chain β-sheet structures along the fibril growth axis (Figure 1D, ribbon diagram). The protein fragments with an α-helix forming propensity could be induced to transition into a β-conformation and form Aβ fibrils (Takahashi et al., 2000).

It has been shown that some ionic self-complementary motifs with oppositely charged residues periodically arranged within a protein sequence are capable of conformational transitions (Farnsworth and Singh, 2000). The unusual changes in the conformation result in the misfolding of a protein/peptide and abrogation of its functions; the protein self-assembles as large aggregates and the high degree of the conformational order of aggregates is referred to as Aβ fibril (Chiti and Dobson, 2009). Peptides can undergo a conformational change resulting in the formation of typical structure termed as cross-β-sheets that break the globular symmetry of the molecules and give rise to linear assemblies of ordered fibers during the process of amyloid aggregation (Ranganathan et al., 2016). Studies have also confirmed that conformational changes in proteins/peptides followed by amyloid aggregation also play a pivotal role in functions like cell signaling and many other physiological processes in the cell (Majumdar et al., 2012). Understanding the structural biology of Aβ peptides produced by the cleavage of amyloid precursor proteins (APP), the transmembrane protein in neurons, is important to decipher the functional consequences (Hardy and Allsop, 1991). Unfortunately, the conformational dynamics of full-length APP is not clearly understood because its amino-terminal is intrinsically disordered.

**FIGURE 2:**
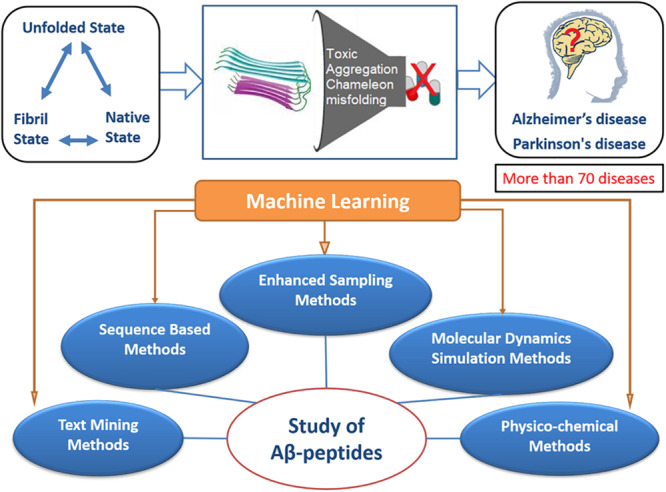
In amyloid formation, flexible sequence fragments of an amyloid precursor protein undergo many structural changes that may cause toxicity, aggregation, and misfolding, which ultimately lead to several neurological disorders, as schematically shown in the upper panel of this figure. In the lower panel, the schematic diagram represents the studies performed by researchers from various disciplines to study Aβ peptides. Among the various methods used, such as sequence based, molecular dynamics simulation, physicochemical methods, etc., the machine learning method could be combined with these methods, providing a better understanding of the mechanism of Aβ peptide aggregation.

Most of the computational studies on proteins with intrinsic disorder rely on the related structures that have been solved experimentally. On the basis of structural models from these experiments, several fundamental questions underlying amyloid formation and its stabilizing interactions have been raised. Amyloid formation is known to involve the construction of fibrils from independent monomer units formed by non-covalent interactions by the stacking of β-sheet structures (Hall et al., 2005). In this paper, we comprehensively reviewed the computational studies performed by researchers from various disciplines to study the Aβ peptides and the main framework is presented as a schematic diagram in Figure 2. Herein, we mainly insist that the reliability of computational studies on Aβ peptides mainly depends on the sampling methods and force fields used. Some of the related physical or theoretical models were also considered to be useful tools for understanding the conformation dynamics of Aβ fibrils. By comparing different simulation methods, we aim to establish clarity on how to choose the appropriate sampling methods and force field parameters for studying certain Aβ peptide molecular systems. Finally, we briefly reviewed the application of machine learning methods for identifying amyloid-like sequences, predicting fibril structures, and developing drug design.

## Aβ Peptides and Neural Disorders

The formation of Aβ takes place in the brain and hence leads to many neurological disorders among which AD is the most prevalent deadly disease in the world. This disease affects most of the functions connected to memory in humans. To cure this disease, we must understand its complex pathology involving the formation of Aβ fibrils. The formation of these pathologies is believed to begin in the hippocampus, where memories are stored and ends in the damage of neurons in the brain. In the amyloidogenic pathway, the formation of excess Aβ peptides blocks cellular signaling leading to the death of neurons (Gaugler et al., 2016). The formation of insoluble amyloid fibrils is linked to many diseases, such as Alzheimer’s disease (AD), Parkinson’s, Huntington’s, amyotrophic lateral sclerosis, prion diseases, type 2 diabetes, and systemic amyloidosis.

To further explore the role of Aβ in neurological disorders, we performed a systematic search on PubMed, a medical bibliographic database using the keyword “Amyloid and neural disorders,” which resulted in 31153 hits. We then identified frequent nearer terms of the domain from the 31153 PubMed abstracts. There are 78 frequent nearer terms found in PubMed abstracts fetched by the PESCADOR tool (Barbosa-Silva et al., 2011). It is interesting to study the numerous neurological disorders, which are connected to Aβ peptides forming fibrils and most of them are related to memory loss. Out of 78 neurological disorders listed in Supplementary Table 1, the PubMed ranker tool suggests that the reports on AD and prion are more frequent in bibliographic databases compared to those on other amyloid diseases (Fontaine et al., 2009). Herein, we discuss the involvement of Aβ peptides in the aggregation/misfolding process in causing two most frequently occurring diseases, namely AD and prion disease, respectively.

Multiple reports in PubMed deal with the theoretical and experimental aspects of the amyloid peptide involved in AD and its principal pathogenic components like fibrils and plaques. Although advances in medical technologies lead to an improvement in the health status of older adults, many aged populations suffer and lose their lives to chronic diseases. Among those listed, Alzheimer’s type of dementia affects the independent functioning of aged individuals and eventually leads to death. Dementia is a progressive neurological disorder that leads to a loss or decline in memory. There are different forms of dementia depending upon disorder in the functioning of organs. AD’s type of dementia is most popular and its symptoms include difficulty in remembering names and recent events, disorientation, and confusion. The progression of each stage of AD varies in individuals, and physical immobility, non-communication, and death are unavoidable in the final stage. The levels of cerebrospinal fluid in Aβ fragments and of hyperphosphorylated or total tau protein are the most widely used diagnostic biomarkers; however, early diagnosis of AD type dementia is still a challenging task (Nakamura et al., 2018).

Very recently, Ma et al. (2018) reported an effective way to modulate the conformation of Aβ peptide for proper protein folding to prevent AD. A modified polyoxometalate has been used as a modulator to disaggregate the β-sheet-rich Aβ aggregates that are crucial to the etiology of the disease. The advantage of using modified polyoxometalate is the capability of crossing the blood-brain-barrier without disturbing the cerebral metal homeostasis and the conformation of other proteins in the neuronal cells. The above study on the mechanism of modulation is powerful and believed to be applicable in preventing the misfolding of other proteins that destroy brain cells and tissues. Considering the prevalence of AD, several researchers have questioned the genetic link of Aβ production in this disease but the answer has remained elusive (Sun et al., 2017). Because other molecules, such as Tau, are also involved in signaling cascades in the brain, a miscommunication between these molecules affects the signaling and leads to neurological disorders. There is still an argument among researchers on whether Aβ peptide is the right molecular target for AD. However, it is understood that the formation of Aβ peptide fibrils in the brain is an important event and is believed to be the culprit for the disorder, and therefore, an understanding of the mechanism of fibril formation is essential for combating neurological disorders. In recent studies, the formation of neurotoxic pores by the Aβ aggregates in the lipid rafts has been suggested to be the main reason for the failure of therapeutics in the past (Arbor et al., 2016).

Besides the monomer and fibril states of the peptide, Aβ oligomers, which are intermediates populated during the aggregation process, were more toxic than peptides in the other states. Aβ oligomers are a key pathogenic source in many neurodegenerative diseases (Bieschke et al., 2012) and have well-organized structures to perform their pathological functions. Amyloidogenic proteins or peptides can form pore-like oligomeric structures by aggregating in the lipid bilayer environment and disrupting membrane permeability (Lashuel et al., 2002). For example, the atomic structures of two overlapping 11-residue fibrillar segments of the human islet amyloid polypeptide have been experimentally shown to form β-sheet-rich aggregates with contrasting cytotoxicity profiles (Krotee et al., 2017). Multiple reports on the formation of Aβ oligomers have revealed their high toxicity but the cause for it remains to be uncovered. Unfortunately, all the drugs and antibodies targeting the production, aggregation, and toxicity of Aβ oligomers have failed because of a poor understanding of the transition in the conformation of the oligomeric state to the fibrillar state (Doig et al., 2017). Uncovering the inter-relationship of various Aβ oligomeric states is crucial for understanding their toxicity and can help in the development of therapeutics against the diseases they cause.

There are several approved drugs, such as Donepezil, Rivastigmine, Galantamine, and Memantine, in the market for treating AD but these have been associated with adverse effects. Few antioxidants, such as nicotinic receptor agonist and PPAR gamma agonist, are under evaluation as therapeutic agents. Galantamine is a tertiary alkaloid extracted from the bulbs of the plants in the *Amaryllidaceae* family. It has a dual mechanism of action in the brain and improves cognition, behavior, and function (McShane et al., 2019). This drug competitively inhibits acetylcholinesterase and allosterically modulates presynaptic and postsynaptic nicotinic receptors, which results in neuroprotective effects. Interestingly, another nicotinic receptor agonist, EVP-6124, is in the Phase 2 trial. Although immunization could be a better way to prevent AD, it does not prevent progressive neurodegeneration and, hence, effective methods to treat AD are needed.

In the past two decades, continuous efforts have been made globally to develop drugs for AD, but progress has not been significant. Among the failed efforts, there have been cases of many monoclonal antibodies, such as Bapineuzumab, Solanezumab, Crenezumab, and Gantenerumab, which were designed to block or eliminate the Aβ peptide. All these drugs failed due to low efficacy or serious side effects. Avagacestat and Semagacestat are two drugs that cure amyloid diseases by targeting gamma-secretase. Tau is also believed to be a potential target in the treatment of AD because of its relationship with aggregation and disease progression (Mehta et al., 2017). There are many arguments regarding the failure of drugs that target amyloid diseases. These include: (1) whether the current amyloid-related targets are really good therapeutic targets? (2) Whether the failure to diagnose amyloid diseases early warrants the designing of effective drugs? (3) Whether enough time is given for drug development, especially for such long-term diseases? The continuous failure of a candidate drug that targets Aβ is due to the difficulty in designing a drug that prevents the aggregation of amyloid peptides, which have dominated the AD for decades.

Another family of diseases closely associated with conformational changes is Prion diseases that are fatal neurodegenerative conditions originating spontaneously, genetically, or upon infection. Conformational changes in prion proteins from the normal to a disease-associated form are considered central to the pathogenesis of prions. This phenomenon is still poorly understood because of its existence in multiple forms. Although progress has been made toward an understanding of the structural consequences of prions, a major gap exists in knowledge about how conformation changes are related to the death of neurons (Zhang et al., 2018). Because the pathogenesis of prion diseases arises from multiple complex pathways, it is very difficult to distinguish the normal and disease forms. In short, it is postulated that an understanding of how conformational changes in prions are associated with the death of neurons may help in the design of drugs to target multiple processes involved in neurodegeneration. Moreover, an understanding of the control parameters for pathogenic changes in the conformation may help reverse the fatal switching to the normal form. It is believed that basic research on conformational dynamics of a protein can help in the identification of more control parameters, which may ultimately lead to a better understanding of the pathology (Moulick et al., 2019).

Understanding the pathogenetic mechanism of prion diseases is difficult because these diseases can be caused by multiple factors, which could be genetic, sporadic, and acquired. To understand these mechanisms, several research groups around the globe, especially those at the University of California, Medical Research Laboratory and Scripps Research Institute, work specifically on prion proteins to identify small molecule drugs that can be used to treat prion diseases (Kawasaki et al., 2007; Lu et al., 2013). Although none of these groups has found any effective small molecule suitable for clinical trials in humans, they have made promising progress. For example, small molecules that work well on mouse prion strains but not on human prion strains have been discovered. Thus, it remains a challenge for researchers to propose models to understand the conformational dynamics of prions, which would ultimately lead to the discovery of drugs for prion diseases.

## Theoretical Models to Study Aβ Peptides

Considering the importance of conformation dynamics of Aβ-peptide in neural disorders, an understanding of the mechanisms of folding and aggregation of these peptides in the cell is of interest to researchers. In this section, we discuss approaches based on knowledge of physics developed in the past two decades to understand the formation of amyloid fibrils and amorphous β-aggregation. The amyloid-forming peptides have been analyzed by physicochemical, knowledge-based energy potentials, molecular dynamics, and machine learning techniques. The knowledge gained from these studies has particularly been used to design novel amyloid-forming fragments to understand the mechanism of amyloid formation. In most of the computational studies, researchers aim to discriminate amyloid-forming and non-amyloid forming peptides using sequence and structural features (Thangakani et al., 2013). Because the probability of a peptide forming amyloid fibrils also depends on experimental conditions, researchers use molecular dynamics to study peptides under different conditions, such as temperature and pH. Herein, we discuss the powerful computational approaches that have been used in recent years to gain insights into the formation of amyloid fibrils.

Several investigations on the structures of Aβ peptide have confirmed that all types of fibrils have a common core structure made up of a helical array of β-sheets stabilized by hydrogen bonds (Kirschner et al., 2006). All amyloid fibrils have diameters around 100Å and appear as hollow cylinders or ribbons (Serpell et al., 1995). Based on some strong hypotheses like the amyloid cascade hypothesis and energy landscape theory proposed by physical chemists and biologists, mathematicians have provided a discrete model to understand the mechanism of aggregation of amyloids (Hardy and Higgins, 1992; Pallitto and Murphy, 2001). It is believed that mathematical models provide a clear mechanistic understanding of the growth of amyloid fibrils (Puri and Li, 2010). Lee et al. (2007) presented a three-stage mathematical model based on protein misfolding, nucleation, and fibril elongation supported by the features of homogeneous fibrillation responses.

**FIGURE 3:**
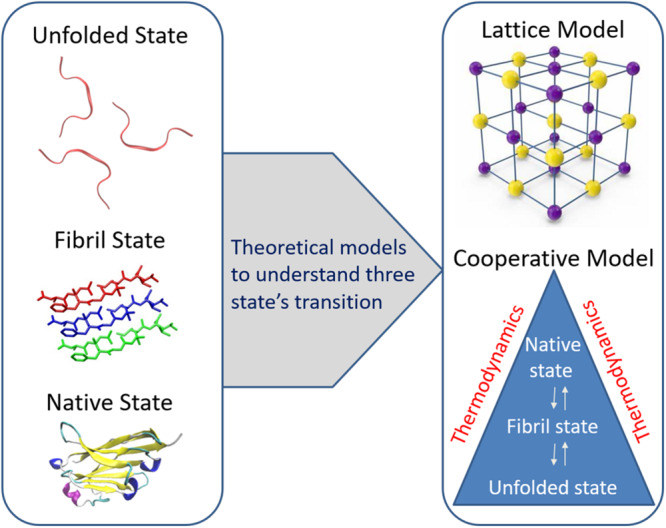
Two important theoretical models such as lattice model and cooperative fibril model respectively are presented to understand the capability of polypeptides to convert from their monomeric native state to amyloid fibrils. Reproduced from Chen et al. (2018) and Espinoza Ortiz and Dias (2018).

Among all the physical models, lattice and cooperative fibril models have been specifically developed to understand the capability of polypeptides to convert from their monomeric native state to amyloid fibrils (Figure 3). Lattice models have previously been used successfully to understand the transition of a protein from its unfolded state to its folded state (Coluzza et al., 2003). Likewise, Abeln et al. (2014) proposed a simple lattice model to represent the formation of backbone hydrogen bonds as observed in amyloid fibrils and their model allows the modeling of the geometric properties observed in β-strands at less computational costs. The model uses four important parameters of amino acid residues in the peptide, namely their position, secondary structural state, side-chain direction, and amino acid type, to simulate the different transition states of the peptide. Some off-lattice models (consider hydrogen bonds and amino acid interactions to study folding and aggregation), which are computationally expensive than the lattice models, have also been proposed to simulate the conformational transition of monomeric peptides to non-specific aggregates (Combe and Frenkel, 2007).

By using the popular cooperative model (Hansen et al., 1998) describing the folding and unfolding transitions of proteins, a modified cooperative model has been described in which polypeptides are allowed to adopt amyloid-like fibrillar structure and this model describes the formation of both native and fibril bonds in a cooperative manner using three Hamiltonian terms of unfolded, fibril, and native states (Espinoza Ortiz and Dias, 2018). These Hamiltonian terms were used to study the conformational transitions from one state to another. The thermodynamic cooperativity between the three transition states considering the temperature function has provided key insights into the understanding of Aβ fibril formation. The key feature of the cooperative model when compared with other models used in studying the formation of fibrils is independent of protein structure and mainly relies on thermodynamic parameters.

From the literature, it is clear that considerable efforts have been made in the past three decades to understand the folding of the Aβ peptide and fibril formation. However, independent theories have been developed to describe the mechanisms underlying amyloid folding (Nasica-Labouze et al., 2015). Many other macromolecular interactions are known to influence the process of aggregation and misfolding. Multiple reports of the association between β-amyloid peptide, α-synuclein, and tau proteins are available in the literature and have been reviewed in detail (Marsh and Blurton-Jones, 2012). The interactions between the Aβ peptide and α-synuclein have been proven by various research groups but very few reports are available about the mechanisms through which these molecules interact together in the progression of disorders in humans. Also, it was shown that the α-synuclein and tau proteins can indirectly influence the effects of Aβ peptide, modulating phosphorylation, and aggregation in the cell. The roles of the above two neuronal cell proteins in affecting the structure, folding, and function of the Aβ peptide have previously been reviewed (Peuralinna et al., 2008). The sequence and structural information for amyloid fibrils are compiled in an online database called AMYpdb, which can be accessed by metAmyl, a software for the prediction of aggregation-prone regions (Pawlicki et al., 2008). Consequently, several analytical methods have been proposed based on the sequence and structural features of short amyloid-forming peptides, which are aggregation-prone regions. The protein scientists researching at the interface of physics, chemistry, and biology work together to understand the mechanism and to identify important features responsible for the formation of amyloid fibrils, which will lead to the development of drugs for many neurodegenerative diseases (Thangakani et al., 2012).

## Molecular Dynamics to Study Aβ Peptides: From Coarse-Grained to All-Atom Model

Besides the physical models, more accurate computational methods are necessary to explore the protein structure and its mechanism of folding/misfolding. Protein structures could be represented using different models such as the one-bead residue model or the electron cloud density matrix model. The basic unit is each atom in the all-atom (AA) force field, while the coarse-grained model represents each residue by one or several beads with united atoms. In a sense, the lattice model can also be regarded as a one-bead model. Distinguishing between the theoretical models and the coarse-grained (CG) models is difficult, as the complexity of theoretical models is increasing. In most CG models, an imprecise criterion is derived based on the number of amino acid types and energy terms such as the native-contact energy term (also known as the Go-term). Most of these models typically consider all 20 amino acid residues with less emphasis on paired interactions. In this section, we have provided a brief summary of the MD studies performed on Aβ peptides that have used CG and AA models. In the past decade, several MD simulation studies on amyloid peptides have been performed including CG MD (Chiricotto et al., 2017) and AA MD (Nasica-Labouze et al., 2015) simulation studies and those that have used enhanced sampling methods; however, we have mainly focused on the progress made in the past 5 years. In this brief review, we aim to provide our recommendations as to how to choose the proper model and method for understanding a certain amyloid peptide system. Sampling methods are one of the most fruitful and active research areas in amyloid-peptide studies. To keep each section concise, we will discuss enhanced sampling technology and its application on Aβ peptides in the next section.

In the last 20 years, several CG models have been applied to study amyloid peptides and its aggregation mechanism, such as OPEP, PRIME, MARTINI etc. Additional information about the introduction and comparison of various CG models could be found in a recent review paper (Singh and Li, 2019). Considering the benefits derived from computational efficiency, CG models could be used to study the oligomerization and fibrillization of amyloid peptides. Rojas et al. used the CG united-residue (UNRES) force field to examine Aβ (9-40) fibril growth on template presenting (Rojas et al., 2017). The UNRES force field can correctly determine the dock-lock mechanism. Furthermore, the “stop-and-go” mechanism of fibril growth can also be observed in their MD simulation. The non-native hydrogen bonds formed between the monomeric chains with the template in the “docking stage” are long-lasting. Another famous CG model, the PRIME20 force field combined with discontinuous molecular dynamics (DMD), has been used for studying the crowder effect of Aβ fragment, wherein 192 Aβ (16-22) peptides and crowders are placed together in boxes with various diameters. MD results revealed that the Aβ fragment aggregation from oligomers to fibril enhanced by the crowders, which is consistent with experimental work (Latshaw et al., 2014). Furthermore, several factors pertaining to the crowders such as surface area, volume fraction, and size have a direct effect on the aggregation rate. Interestingly, the formation of Aβ oligomers of a specific size is also regulated by crowder diameter, which implies that the transition pathway may be influenced through a complex mechanism in the crowded environment inside cells. Zheng et al. (2017) predicted the propensity of amyloid formation and fibril topology using a CG-optimized folding landscape model, which has already provided insightful knowledge about protein structure prediction, protein association, allosteric mechanism, and protein aggregation. In the AWSEM (Associative memory, Water mediated, Structure, and Energy Model) model, each residue is designated by three beads. In the AWSEM model, the oligomerization of Aβ (1-42) and Aβ (1-40) in the monomeric to the octameric form is studied (Zheng et al., 2017). The Aβ42 has a more diverse structural range in the tetrameric form, and lower free energy barrier than Aβ40 during fibril formation. Further, this model uses the same principles for detecting amyloidogenic segments and predicting the relative orientation of the amyloid β-strands in the fibril core (Chen et al., 2018). The advantage of the AWSEM model is that it can perform high-resolution *de novo* structural prediction and can produce better structural models than the already existing methods. Recently, Wang et al. (2019) developed a relatively precise CG model to study intrinsically disordered proteins (IDP), a large group of proteins that lack ordered structures, using thermodynamic parameters. The authors reported the solubility of the Aβ peptide predicted by modeling the thermodynamic phase diagram and their results were in agreement with the morphological data of fibrils previously determined by electron microscopy.

**FIGURE 4:**
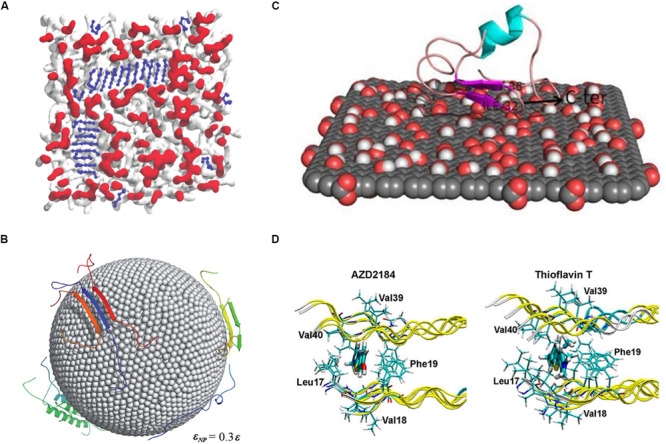
**(A)** MD simulation using the MARTINI-derived force field to examine membrane-induced Aβ_16__–__22_ fragment aggregation. **(B)** Investigating the Aβ peptide aggregation on nanoparticle surface using the two-bead CG model. The surface atoms of the nanoparticles are shown as spheres and proteins as cartoon representations. **(C)** An all-atom MD study of monomeric Aβ_1__–__40_ binding on the surface of graphene oxide nanomaterials. Graphene atoms are shown in gray, while the oxygen and hydrogen atoms are represented in red and white. **(D)** The study of Thioflavin T and AZD2184 binding with the Aβ_1__–__42_ fibril performed using molecular docking and molecular dynamics simulation methods.

When compared with AA models, CG models offer the advantage of studying extremely large biomacromolecular complexes. Sahoo et al. (2019) performed MD simulation with the MARTINI-derived force field to examine membrane-induced Aβ (16–22) fragment aggregation (Figure 4A). In their study, 48 Aβ (16–22) fragments were placed on 13.4 × 13.4 nm^2^ membranes. Two types of membranes that have different lipid head group charges were studied: zwitterionic POPC (1-palmitoyl-2-oleoyl-sn-glycero-3-phosphocholine) and anionic POPS (1-palmitoyl-2-oleoyl-sn-glycero-3-phospho-L-serine). The POPC lipid membrane led to faster fibrillation of Aβ peptides than the POPS membrane, which implies that various pathways are regulated by the lipid head group for peptide absorption into membranes. Radic et al. (2015) studied Aβ peptide aggregation on nanoparticle (NP) surface by using DMD combined with the two-bead CG model (Figure4B). In their model, the NPs were represented by two layers close-packed all-atom sphere and the diameter of the NPs was 10 nm. By regulating the non-specific attraction strength of NPs and the NP/peptide ratio, they found that low, non-specific interaction enhances Aβ peptide aggregation but the high attraction prohibiting it. This result has thus provided a key insight regarding the potential of Aβ peptide aggregation-inhibiting, NP-based drugs for AD treatment.

Previous studies performed using the AA MD model have reported its advantages, such as high accuracy and wide applicability. The AA MD model is similar to classical protocols in terms of fibril stability, interaction of ligands with amyloid fibrils, and fibril aggregation modulator, such as metals, pH, membrane, etc. For example, several AA MD based studies have discussed the effect of ligands on Aβ peptide aggregation because they are related to direct drug designing. The model is now extensively used for studying the active site, binding affinity, and binding mechanisms of ligands with amyloid fibrils. In principle, the MD simulations of the interaction between ligands and amyloid fibrils reported in these studies have followed the essential assumptions for understanding the binding mechanism. To identify the correct binding sites, factors such as force field of ligands, and location of the binding site should be duly considered. Once the binding positions are found, MD simulations can help in recognizing the important residues interacting with the ligands and in determining the effect of a ligand on amyloid formation. We have discussed some recent studies in this context as follows: He et al. (2017) performed MD simulation to identify the effect of binding of a fluorescent amyloid-binding probe, amino naphthalene 2-cyanoacrylate (ANAC), on three different amyloid fibrils, namely Aβ40, Aβ42, and prion proteins (He et al., 2017). The parameters of ANAC molecules were calculated with quantum chemistry software, Gaussian09, and ligands were placed more than 2.5 nm away from the fibrils in the beginning, to search for the binding sites. The results showed that ANAC ligand, which contains both aromatic and hydrophobic groups as an amphipathic molecule, was preferentially bound to the aromatic side chains and positively charged residues in the corresponding parts. The binding modes showed differences in the three fibril models, implying the selectivity of amyloid fibrils for this probe. Another similar work investigated two important amyloid imaging tracers, which could be used for early diagnosis of Alzheimer’s, using all-atom MD simulations (Figure 4D) (Kuang et al., 2015). In this case, the binding site of thioflavin T and AZD2184 on the Aβ42 fibril structures was determined using docking methods and the mechanism was studied using MD simulation. The inside region of two β-sheets was found to be the core binding sites for both the compounds, and Met35 was highlighted as an important residue. Interestingly, AZD2184 showed better binding affinity than thioflavin T, which is consistent with the experimental results. Another MD simulation study focused on curcumin, a well-known inhibitor of Aβ (Awasthi et al., 2018). The stability of the helical structure of wild-type Aβ42 was compared to those of two familial mutants, namely A2V and A2T. In the control runs, the harmful mutant (A2V) had higher stability than the wild type and protective A2T mutant. However, the root mean square deviation (RMSD) results showed an opposing situation after the interaction of curcumin with the peptides; the A2T mutant becomes more stable than A2V. The binding energy of curcumin provided a clue about the alteration in the stability because the A2T mutant had a higher binding affinity than that of the A2V mutant, which could lead to less flexibility of the overall structures. As we have mentioned, most of the MD simulation studies on ligands follow the basic procedure starting from the identification of binding sites and then elucidation of the mechanism. It should always be borne in mind that MD studies on drugs against amyloid fibrils are sensitive to the amyloid models because most of the amyloid peptides are found to exist in more than one fibrillar structures *in vivo*/*vitro* (Tycko, 2011). The correct conclusion about the mechanism of ligand binding or regarding the effect of drug relies on the use of proper simulation protocol, selection of the amyloid model, among other factors.

During the aggregation of peptides, several environmental factors, such as membrane, metal ions, and pH, influence and regulate the oligomerization or fibrilization process. The identification of the key factors promoting the aggregation or formation of toxic species would improve the understanding of the pathogenic mechanisms of amyloid diseases. Based on studies on the peptide-only system, these regulatory factors are taken into account in MD simulations and explicitly represent them as molecules or implicitly as certain interactions. Hence, each type of factor requires a specific method and skill. For example, in the case of the membrane, adding lipid molecules explicitly into the simulation system would always lead to a huge number of atoms and slow sampling efficiency. Devarajan and Sharmila (2014) conducted an all-atom MD study on the Aβ peptide with GM1 ganglioside in implicit DPPC lipid membrane and observed the conformational changes in the protein backbone due to the influence of ganglioside as well as of the hydrogen bond interactions between the active site amino acid residues of Aβ42 and the GM1 head group moieties. Lu et al. (2018) investigated the effects of constant electric field on the Aβ 29–42 dimer inside a membrane using all-atom MD simulations. They added a 20 mV/nm electric field across the lipid bilayer and examined if it would affect the membraned-embedded Aβ segments. The results revealed that the different secondary structures of peptides reacted differently upon the application of an electric field, as α-helix peptides converted from the Gly-out to Gly-in state, and β-sheet peptides changed the kink and tilt angles at Gly33 and Gly37. Overall, MD results support the conclusion that the conformational distribution of transmembrane amyloid peptide would shift in the presence of an electric field in neuronal cells. A similar scenario appears for the surface of nanomaterials; for example, a recent MD study was conducted on the influence of graphene oxide surface on Aβ peptides (Figure 4C) (Baweja et al., 2015). The simulation results were in agreement with the experimental results showing that graphene oxide could decrease the β-strand propensity of the Aβ peptides. Furthermore, a comparison between graphene oxide and reduced graphene oxide revealed that the latter acts as a better inhibitor of fibril formation because it has extra van der Waals interactions than the former, leading to better adsorption of the Aβ peptide on the surface and enhances conformational transitions.

Metal ions affect the kinetics of Aβ aggregation, with the most significant effect on the nucleation phase. For example, zinc and copper affect the population and/or the type of aggregation intermediates formed. Unlike in the case of membranes, one should be very careful while dealing with the force field representing the transition metal ions appropriately and accurately. Using MD simulations, Duane et al. (1987) showed that Zn ions promote Aβ aggregation via a shift in the population of polymorphic states. With the help of MD simulation, it was reported on how metal ions play a role in the formation and stability of Aβ oligomers and fibrils (Lee et al., 2018). It has been shown that zinc and copper ion increase the stability of Aβ oligomers, whereas other metal ions reduce the stability of Aβ fibrils. In addition, it was found that zinc ions could destabilize the fibril structures more effectively than copper ions. The study by Miller et al. sheds light on the role of the metal ions, known as toxic agents, in stabilizing the amyloid oligomers, which is consistent with clinical observations that high concentrations of metal ions are found in patients suffering from neurodegenerative disease. Another study used the ligand field molecular mechanics simulation to model the interactions of copper and platinum with the Aβ 1–42 peptide monomer (Mutter et al., 2018). The results of molecular dynamics simulation over several microseconds were compared to analogous results for the free peptide. Significant differences in structural parameters were observed, between both the Cu and Pt bound systems as well as between free and metal-bound peptide. Both the metals stabilized the formation of helices in the peptide and reduced the content of β secondary structural elements compared to that in the unbound monomer.

The CG or AA model has its own applications. The CG model can be applied to larger systems that contain thousands of residues or with bilayer membranes. The sampling efficiency is good enough to handle long timescale conformational transitions, such as fibril formation. However, the CG models are greatly limited in estimating accurate interactions, such as the effect of ligands or metal ions on Aβ peptides. This does not mean that these models are not accurate at all but the cost versus accuracy equation is not balanced. For example, one of the CG-model used in a study determined the interactions between an inhibitor and U-shaped Aβ(17–36) protofilament DMD combined with the PRIME20 force field (Wang et al., 2017). Four inhibitors were tested: vanillin, resveratrol, curcumin, and epigallocatechin-3-gallate (EGCG). The EGCG showed the best inhibitory effectiveness with the Aβ fragment, which is consistent with experimental results. However, similar to CG models, the protocol that generating bond and energetic parameters from AA MD runs are relatively cumbersome and have poor versatility. Compared to the all-atom force field, the small ligands in the CG model reply on empirical parameters, which hamper the reliability of the results. Compared to the CG models, the AA models include the computational cost required to achieve higher accuracy. With the improvement in computational performance, the AA models have become a better choice in several scenarios such as determining the structure of the membrane-peptide complex. However, based on the comparison shown in Figures 4A,C, it is clear that the CG and AA MDs would have different system sizes and timescales on the membranes and numbers of peptide chains. Researchers must consider that each model has its own limitations. They should hence have a clear understanding of which simulation methods will help one pick the proper models for a certain scenario and draw a balance between cost and accuracy. In the next section, we have discussed several enhanced sampling methods that are particularly suitable for IDP, including Aβ peptides.

## Application of Enhanced Sampling Methods on Amyloid Peptides

The most important characteristic of amyloids is that they are easy to aggregate and can form oligomers and fibrils. With the progress in research, scientists have found a change in the conformational behavior during the aggregation process, and that the choice of different conformations has affected the mechanism of aggregation of the Aβ peptide. In addition, some studies have found that metal ions can induce the aggregation of Aβ (Atwood et al., 1998; Hindo et al., 2009; Li et al., 2016). Owing to the high insolubility of Aβ aggregates, it is very difficult to experimentally decipher the process of aggregation of antibodies at the atomic level. Although several new biophysical techniques are being used to develop a better understanding of the structure and aggregation of Aβ proteins, experimental studies alone are not sufficient enough because they produce time- and space-averaged results. The challenges and limitations inherent to the current set of experimental techniques for studying these polymorphic, aggregation-prone Aβ monomers have encouraged many researchers to use a wide variety of computational properties of these peptides. By exploring different time and length scales, computerized simulations can complement experimental studies. Simulations are very challenging due to the intrinsic flexibility and heterogeneous ensemble of the monomeric amyloid peptide monomers and oligomers and the impact of various regulatory factors. Conventional MD methods are stretched when dealing with the high level of complexity of the conformational spaces of oligomers and various meta-stable states with close free energy.

As a comparison, the microcanonical MD could deal with ligands binding with amyloid fibrils, but, if one studies the conformational space of the Aβ monomer, the MD simulations would be trapped into local minimal at room temperature quickly and the efficiency for seeking various states of flexible peptide chains would be low. The target of all enhanced sampling methods is to improve sampling efficiency for exploring the conformational spaces; in most cases, this means a general ensemble instead of microcanonical ensemble. Since most of the amyloid peptides have an intrinsic disorder and do not have a unique structure in their monomeric state, enhanced sampling methods are widely used in studying the structure of Aβ peptides. A list of popular enhanced sampling methods and its brief introduction could be found in a previous review, which includes replica-exchange molecular dynamics (REMD), accelerated molecular dynamics simulations (aMD), meta-dynamics, umbrella sampling, etc. (Bernardi et al., 2015; Morriss-Andrews and Shea, 2015). In this section, we are more concerned with the application of sampling methods in amyloid peptides and not the details of the method; therefore, the content is listed according to the molecular system and not by the methods.

The first and direct application of sampling methods is determining the conformational space of monomeric or oligomeric Aβ peptides, as well as the mechanism of oligomerization. A series of studies predict the transient conformations of Aβ hairpins with REMD (Friedman et al., 2009; Adlard and Bush, 2018; Lane et al., 2018). Their results demonstrated a structural similarity to the models of highly ordered aggregates, suggesting that these hairpins may act as seeds for Aβ assembly. Temperature-REMD (T-REMD) and Hamiltonian-REMD (H-REMD) are widely used for the study of simulations of aggregation from random states (Côté et al., 2012; Ayton et al., 2013; Mutter et al., 2018) and the interaction of non-Aβ amyloid component and Aβ. Meng et al. (2018) employed single-molecule Förster resonance energy transfer spectroscopy with site-specific dye labeling using an unnatural amino acid and REMD simulations to investigate conformations and dynamics of Aβ isoforms, Aβ40 and Aβ42. The results show that both the peptides populate configurations consistent with random polymer chains, with the vast majority of conformations lacking significant secondary structure, giving rise to very similar ensemble-averaged FRET efficiencies that both Aβ40 and Aβ42 populate an ensemble of rapidly reconfiguring unfolded states, with no long-lived conformational state distinguishable from that of the disordered ensemble. One of the REMD simulations focused on the Taiwan mutation, revealing that the β-content is decreased in the Aβ40 and Aβ42 monomers, in agreement with the results of experiments wherein it was shown that the D7H mutant slowed the formation of fibrils and the mechanism was related to the salt bridge Glu23–Lys28 (Truong et al., 2014).

Although the oligomers of the amyloid peptide have been studied using MD simulations for more than 15 years, performing sampling for the transition from the oligomeric state to the fibrillar state and identifying the toxic oligomers is still challenging. Hashemi et al. (2019) performed 3 ms aMD simulations for analyzing the dimerization of Aβ40 peptides. aMD is an enhanced-sampling method in which the potential energy terms are simplified into dispersed states and the energy barriers between adjacent energy basins are reduced (Hamelberg et al., 2004). The dimer structures are classified by cluster analysis and the inter-chain interactions are mainly located in the regions of residues 5–12, 16–23, and 30–40. Another work studied the dimerization of the Aβ polypeptide using REMD, with each replicate lasting for 300 ns (Watts et al., 2018). The analysis of intra- and inter-chain residue distances showed that although the individual chains were highly flexible, the dimeric system stays in a loosely packed antiparallel β-sheet configuration with contacts between two CHC regions. Similarly, Van Der Munnik et al. (2018) studied the Aβ 1–42 dimer with REMD. The output configurations were taken from the simulation used in a self-consistent field theory that explicitly accounts for the size, shape, and charge distribution of the amino acids comprising Aβ and all molecular species present in solution. The solution of model equations helps in the prediction of the probabilities for configurations of the Aβ dimer and the potential of mean force between two monomers during the dimerization process. In another study, the REMD simulations of the structure of Aβ 11–40 trimer in the presence of an explicit solvent were determined (Ngo et al., 2017b). The probability of the β-content and amounts of random coil structure in the solvated trimer is in good agreement with the experimental results. Intermolecular interactions in the central hydrophobic cores play a key role in stabilizing the oligomer.

The mechanism of propagation of fibrillar growth is an important and classical topic in amyloid peptide research and the most popular theory is the so-called “dock/lock mechanism.” Recently Schwierz et al. (2016) studied the fibril growth propagation of Aβ9-40 with umbrella sampling and involving multiple processes: fibril elongation by a single peptide at two unequal fibril ends and association of a larger filament. Their results are consistent with those of previous studies that reported docking is much faster than the lock process because of the trapping of long-lasting non-native hydrogen bonds. Moreover, kinetic analysis performed based on position-dependent diffusion revealed that the lock stage fibril growth required a collective motion of water molecules to create a dry interface and the mobility of the involved hydration water showed a 2-fold reduction in the diffusion coefficient. Apart from the seeding effect of the self-elongation of fibrils, the so-called cross-seeding effect, which happens between different species of amyloid peptides, is also important because it is related to the clinical risks of cross infection. Baram et al. (2016) constructed cross-seeding fibril models of the islet amyloid polypeptide 1–37 and Aβ 1–42 peptides and studied the stability of the complex. Interestingly, they generated two kinds of cross-seeding fibrils: one was linked in a single layer and the other was packed into double layers. The single layer showed better stability than the double layer model in most of the cases, which implies that cross-seeding, rather than a combination of different species of protofibrils may occur in alternate fibril elongation. Another extensive REMD simulation study focused on the cross-seeding dimerization of amyloid peptides, IAPP and prion 106–126 fragment (Chua et al., 2016). The highly diverse aggregating complex suggests the formation of highly polymorphic cross-species fibrils or oligomers between the two peptides. Similar to the interactions found in Aβ peptides, hydrophobic interactions including aromatic–aliphatic interactions play a key role in intra- and inter-chain interactions and in the formation of cross-seeding β-sheets. Jose et al. (2014) studied the cross-dimerization effect between Aβ (1–42) and α-synclein (α-syn) (1–95) which is related to Parkinson’s disease with accelerated molecular dynamics simulations (aMD). Their results revealed that the Aβ and α-syn can bind strongly, which is mainly caused by inter-chain salt-bridge and hydrophobic interactions between the non-amyloid component region of α-syn and the hydrophobic core of Aβ.

**FIGURE 5:**
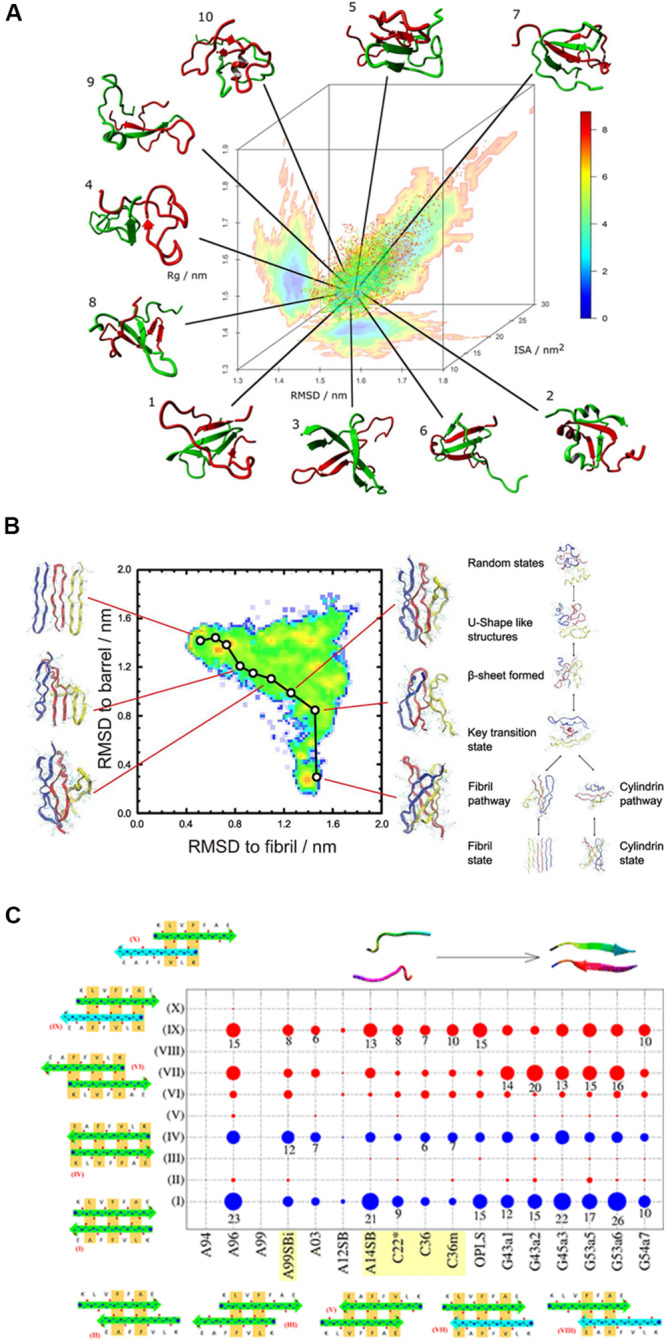
**(A)** A typical REMD simulation that shows the dimerization of the Aβ_1__–__40_ peptide and the free energy landscape that is projected three-dimensionally: radius of gyration (Rg), RMSD from the global average conformation and interaction surface area (ISA). **(B)** Understanding the transition between fibril-like and barrel-like, αB-crystalline segment using the Replica-Exchange-with-Tunneling method. A typical pathway between the two states is represented on the free energy landscape that is projected on the root-mean-square-deviation spaces. The inter-conversion mechanism among the coil structure, fibril state, and cylindrical state is proposed based on the energy landscape. **(C)** Comparison of 17 force fields with the Aβ_16__–__22_ dimer. The conformational populations of the Aβ_16__–__22_ dimer are shown in circles for all the tested force fields. The blue circles represent the in-register antiparallel β-sheets (I and IV) and the red ones indicate the other 8 out-of-register patterns, indicating that the H-bond patterns are shifted and are low in number. The circle size represents the population. AMBER99-ILDN, AMBER14SB, CHARMM22*, CHARMM36, and CHARMM36m are the best candidates for studying amyloid peptides.

The aggregation process is not a one-way polymerization, but a balance of polymorphism and transition between different states. Mudedla et al. (2019) study the transition between the α-helical and β-sheet conformation of Aβ 16-24 segment and its mutant with umbrella sampling simulation. The intermediates are found to be coil-like structures and stabilities by intra-chain hydrogen bonds. Replica-exchange-with-tunneling (RET) (Bernhardt et al., 2016) is a strategy that combines replica-exchange with ideas from hybrid Monte Carlo simulation and molecular dynamics (MC/MD) and enhances the sampling of the transition pathway between different states. Zhang et al. (2017) performed RET simulations to study the conversion between the polymorphic forms of the 11-residue segment of the αB-crystalline peptide (Figure 5B). The results suggest that hydrogen bonds regulate the in-register and out-of-register aggregation states for αB-crystalline oligomers. In the past, H-REMD, in which the general ensemble isn’t coupled with temperature but Hamiltonian, has been used to study the metastable states of Aβ peptide and explorer the conformational space (Côté et al., 2011). As a derivative of H-REMD, RET methods go a further step on sampling transition pathways between different states, for example, fibril-like and barrel-like assemblies in amyloid peptides.

The enhanced sampling methods are also implicated in studying the aggregation modulated by the environment. Ngo et al. (2017a) studied the transmembrane fibril-like Aβ trimer in dipalmitoyl-phosphatidylcholine lipid bilayers with T-REMD. Although the temperatures ranged from 290 to 417 K for 48 replicates, the membrane with the DPPC lipid bilayer was stable during the REMD runs. Two typical hydrophobic core regions, residues 14–19 and 31–37, preferred a β-structure and were inserted into the lipid bilayer whereas the other parts rather favored a random coil structure and were close to the surface of the membrane. Interestingly, one of the important salt bridges, Asp23–Lys28, essential for Aβ40 fibrils in the solvent, is replicated by another polar contact between Asp23 and Asn27 in the membrane environment. Moreover, the binding of the Aβ trimer to the lipid bilayer suggested that the insertion of oligomer into the membrane is a spontaneous process. Compared to electrostatic interaction, van der Waals interaction energy is a predominant factor. In the same year, the same group performed another REMD simulation with a similar protocol to study the membrane environment as well as the mutant effects of the Aβ peptide (Ngo et al., 2017c). The Flemish mutant of Aβ11–40 is placed in a similar transmembrane state. The A21G mutation increases the negative charges near the coil regions in the trimer and consistently there is a larger repelling force from the inside to the outside of the membrane. The oligomers of the A21G mutant showed a lower affinity for the DPPC lipid bilayer and larger flexibility than wild-type in the overall structure, which is consistent with the experiment showing that the mutation leads to lower aggregation and membrane binding rates (Sureshbabu et al., 2010; Lee et al., 2017). Pietropaolo et al. (2015) perform a meta-dynamics simulation to study the complex of Aβ (1–42) with two zinc molecules. The free energy landscapes reveal that with the zinc stabilized the salt-bridge Lys28-Glu22 and Lys16-Asp23 and predominant conformation is hairpin-like in which N-terminal coordinated with zinc ions. Another work studied the Aβ16-22 monomer adsorbed on the gold surface with meta-dynamics simulation (Bellucci et al., 2016). The comparison of conformational spaces of a peptide in bulk water and surface reveals that the gold surface could deplete the fibril-like structure of the Aβ fragment, which is consistent with experimental results.

In conclusion, our review suggests that MD simulations strongly rely on sampling methods or force fields and a comparison of the results obtained using MD simulations and other simulation protocols would be drastically different. Hence, researchers are advised to be careful about how to perform these simulations and compare their results with those of other MD based studies. Force field accuracy is particularly important for understanding the formation of the amyloid peptide aggregates, since the results of sampling methods are very sensitive on exploring several metastable states with close free energy. For example, if the secondary structure propensities of a force field have a preference for α-helix, the REMD simulation of Aβ oligomers would represent much lower probability of forming an inter-chain β-sheet structure. The effect of force fields on Aβ fragment structures has been investigated by several groups. Almost 10 years ago, Wang et al. had compared the effect of OPLS–AA/TIP4P and GROMOS43A1/SPC force fields on conformational spaces in monomeric Aβ 12-28. The β-hairpin structure in the turn region showed considerable differences (Cao et al., 2011). In the same year, another group compared the AMBER99, GROMOS96, and OPLS force fields with the REMD simulation of monomers, dimers, and trimers of Aβ16–22 (Nguyen et al., 2011). This group reported that each force field had a certain bias on secondary structure propensity wherein AMBER99 favored α-helix, while GROMOS96 preferred the antiparallel β-sheet. Somavarapu and Kepp (2015) studied the dynamics of Aβ40 by using various force fields including AMBER, CHARMM, OPLS and Gromos96 combined with water models such as SPC, TIP3P, and TIP4P. Different force fields produce variations in structural ensembles as helices, β-strands, or unstructured ensembles. In short, Charmm22^∗^ and Amber99sb-ILDN provided the best accuracy for experimental NMR chemical shifts. Interestingly, these two force fields prefer β-strands for the Aβ monomer ensembles. Weber and Uversky (2017) tested AMBER99SB and CHARMM22/CMAP force fields for studying the Aβ42 monomer using REMD. The results obtained using both the force fields were comparable with the chemical shift values determined by performing NMR experiments.

Watts et al. (2018) performed an REMD simulation on the Aβ40 dimer using a series of force fields in the AMBER and CHARMM families. The conformational spaces were classified by cluster analysis and projected the energy landscapes in the first 3 dihedral principle components (Figure 5A). They concluded that AMBER99SB-ILDN, AMBER99SB-NMR, and CHARMM36 provide the best accuracy on β-sheet content. Similarly, another group compared 17 force fields with the Aβ 16-22 dimer (Man et al., 2019). They compared the conformational distribution of Aβ (16-22) dimer generated by each force field as summarized in Figure 5C. The blue circles represent the in-register antiparallel β-sheets (I and IV), while the red ones indicate the other 8 out-of-register patterns, which mean that in the out-of-register patterns, the H-bond patterns are shifted and have less H-bond numbers than the in-register β-sheets. The population based proportion is represented by the circle size. The results of this study imply that AMBER99-ILDN, AMBER14SB, CHARMM22^∗^, CHARMM36, and CHARMM36m are the best candidates for studying amyloid peptides. A recent work published by Robustelli et al. (2018) have test several force fields of the CHARMM and AMBER families with a benchmark set of 21 proteins including Aβ40. Interestingly, although most of the force fields treat folded proteins well, none of them can reproduce the secondary structure propensities accurately enough for a disordered protein, including AMBER99SB-ILDN or CHARMM36m. Among these force fields, results obtained using AMBER99SB-disp was in best agreement with previously reported experimental results for most of the proteins in the benchmark set. This force field has been developed by Robustelli et al. (2018) based on the AMBER99SB-ILDN force field using the TIP4P-D water model. The torsion parameters are optimized and small changes in the protein and water van der Waals interaction terms are introduced. The different conformational bias of these force fields reminds us to be cautious when choosing a force field, especially for studying amyloid peptides by using enhanced sampling methods. Generally speaking, the AMBER99SB-ILDN and CHARMM36m always seem to be the safest choices for researchers who want to focus on amyloid peptides and other IDPs, although the AMBER99SB-disp force field is also worth considering for the same purpose.

## Machine Learning Techniques and Its Application on Alzheimer’s Disease

In recent years, machine learning tools and techniques have been used to create more accurate computational prediction models. The identification of fibril-forming peptides is critical to understand the pathogenetic process of various neurological disorders. Machine learning methods and traditional computational algorithms have been used to identify such flexible fragments. These computational models can be classified into two important groups, namely the sequence-based methods that rely on the physicochemical properties of amino acid residues and the structure-based methods that rely on the three-dimensional structure information and energy landscape (Saravanan and Selvaraj, 2012; Sujitha et al., 2014). Several computational methods were developed to predict sequence segments of a protein, which tend to form the fibrillar beta spine form. The better performance of the structure-based approach indicates the critical information for protein fibrillation, which is encoded by the short sequence. Other knowledge-based methods have also been proposed to predict the amylogenicity of a peptide; these include FISH Amyloid (Gasior and Kotulska, 2014), Tango (Rousseau et al., 2006), ZipperDB (Goldschmidt et al., 2010), Pasta (Walsh et al., 2014), AggreScan (Conchillo-Solé et al., 2007), PreAmyl (Zhang et al., 2007), Zyggregator (Tartaglia and Vendruscolo, 2008), CamFold (Tartaglia and Vendruscolo, 2010), NetCSSP (Kim et al., 2009), FoldAmyloid (Garbuzynskiy et al., 2009). A list of currently available machine learning methods is presented in Table 1.

**TABLE 1:** List of currently available programs for amyloidogenicity prediction.

Model	Web server	Approach used
appnn	http://cran.r-project.org/web/packages/appnn/	Feed-forward neural networks
RFAmyloid	http://server.malab.cn/RFAmyloid/	Random forest
FISH Amyloid	http://comprec-lin.iiar.pwr.edu.pl/fishInput/	Site specific co-occurrence of amino acids
PASTA 2.0	http://protein.bio.unipd.it/pasta2/	Pairwise energy potential
AGGRESCAN3D (A3D)	http://biocomp.chem.uw.edu.pl/A3D/	A3D score
TANGO	http://tango.crg.es/	Physico-chemical based
Waltz	http://waltz.vub.ac.be/	Position-specific scoring matrix

There are many reports in the literature that describe machine learning methods, such as the 3D profile method, for accurate prediction of the amyloidogenic segments. Stanislawski et al. (2013) have tested several machine methods to increase the classification efficiency of amyloidogenic candidates, and found alternating decision tree (ADT) and neural network of multilayer perceptron (NNMP) architecture have achieved the best performance, which has an area under curve (AUC) of above 0.96. Based on the physicochemical and biochemical properties of the amino acids, Família et al. (2015) built an artificial neural network that can accurately predict the amyloidogenic propensity. The online server, RFAmy, was reported to achieve an overall accuracy of about 89.19% in the ten-fold cross-validation test (Niu et al., 2018). In another model named AmyloGram, the authors trained the predictors of amyloidogenicity, using n-grams and random forest classifiers, and obtained the highest performance (David et al., 2010).

**FIGURE 6:**
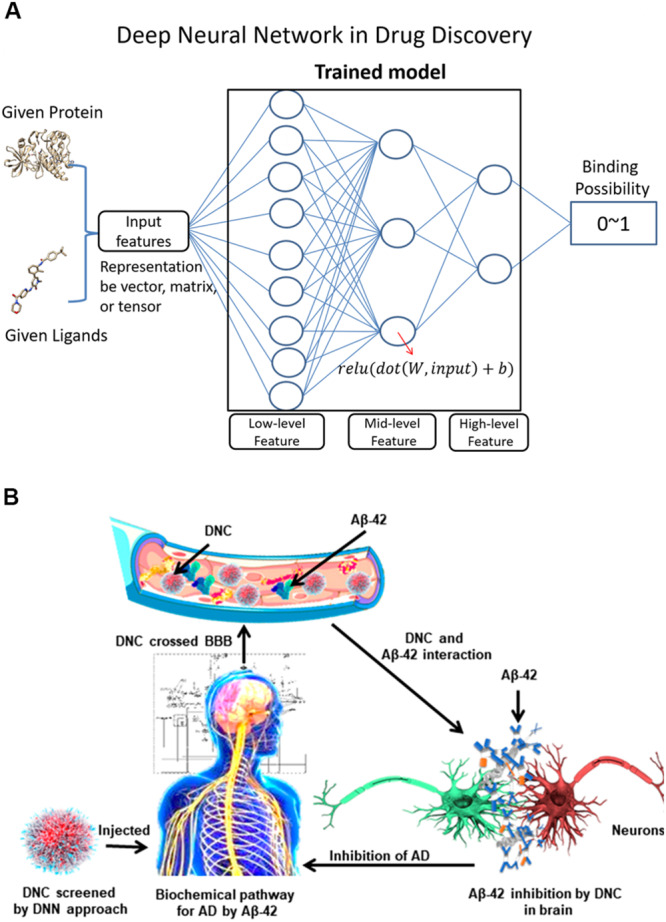
**(A)** Shows a classical deep neural network and its application in protein-ligand binding studies. For a deep neural network, the lower layers usually extract low-level feature, while the higher layers learn the high-level feature. **(B)** Illustrates the screening of a drug-nanoparticle complex (DNC) by using a deep neural network model and DNC interactions with proteins in the pathway of AD. The neurons are shown in red and green colors interactively. Aβ peptides are marked with blue color whereas gold nanoparticles and inhibitors are indicated as orange and gray color respectively.

Machine learning is a useful tool for conducting efficient *in silico* searches over numerous molecular compounds and increases the success rate of discovering potential drug candidates (Figure 6A). Some of the proposed Aβ-related targets are involved in the generation of Aβ, which begins with the β-site APP cleaving enzyme that cleaves the APP, followed by γ-secretase that makes the second cut to produce Aβ. The α-secretase enzyme can cut APP at a different location, preventing the formation of Aβ. Therefore, the most popular inhibitors are BACE1 and γ-secretase. Subramanian et al. modeled the binding affinities of human BACE-1 inhibitors using multiple *in silico* ligand-based modeling approaches and statistical techniques (Subramanian et al., 2016). The results showed that machine learning methods with canvas descriptors resulted in robust classification accuracy and exhibited superior performance compared to the traditional Bayesian techniques. Quantitative regression models suggest that the use of canvas descriptors can achieve better statistical accuracy similar to 3D field-based techniques that often require molecular alignment of diverse chemical scaffolds in one universal chemical space. Aswathy et al. (2018) identified the structural and physicochemical requirements for the potential inhibition of Aβ aggregation, and molecular docking analyses of the representative inhibitors were performed to determine the binding modes of inhibitors at the active site of the protein. They used a random forest based model to test the activity of novel chemical entities and to screen the newly designed molecules. Kaushik et al. (2019) attempted to discover potential inhibitors against Aβ-42 using an *in silico* deep neural network approach (Figure 6B) (Kaushik et al., 2019). They screened PubChem compounds library and found wgx-50 as a potential inhibitor of Aβ-42, the synergistic effects of with gold nanoparticles induced significant inhibition of Aβ 1-42 relative to that induced by wgx-50 alone. Wang and Ng (2019) developed five different machine learning models ranging in complexity from linear regression to a deep neural network. The deep neural network trained specifically on BACE ligands performed best for affinity prediction (Wang and Ng, 2019). Current applications of deep learning in drug development have the potential to facilitate the development of a drug for AD because of several reasons. It is possible (1) to develop a deep learning model that can remove the pan-assay interference compounds (PAINS) (Gilberg et al., 2016) and other unwanted compounds before they enter the experimental stage by learning the approved drug dataset; (2) to develop a highly accurate model to identify the protein–ligand binding and non-binding complex or to estimate the protein–ligand binding affinity by learning from the available experimental protein–ligand complex data; (3) to use deep learning model to predict the early stage of AD; (4) to use the generative adversarial networks (GAN) model to learn about the known ligands of an AD drug and generate compounds with similar synergistic effects of wgx 50 AuNPs complex, indicating its drug potential for AD.

Researchers have effectively used machine learning techniques to classify the status of the AD by learning the scanning pictures of the brain and used deep learning to predict the cognitive decline by brain metabolism and amyloid imaging (Choi and Jin, 2018). Ko et al. (2019) identified multivariate cost-efficient markers for Aβ positivity among non-demented individuals using 10-fold cross-validation of an adaptive least absolute shrinkage and selection operator (LASSO) model. The model showed AUCs of 0.754/0.803/0.864 for the mild/moderate/severe change group, respectively, which demonstrates that machine learning based multivariate neuropsychological assessment and demographic measures are possible ways for predicting the abnormal level of Aβ in non-demented people. Automatic identification of subjects that are most likely to exhibit rapid cognitive decline is very important for effective treatment of neurodegenerative diseases, such as AD. Recently, Choi and Jin (2018) developed a convolutional neural network based automatic PET-image interpretation system for accurate prediction of future cognitive decline in patients with mild cognitive impairment. A deep convolutional neural network (CNN) was trained using 3-dimensional PET volumes of AD patients and normal controls as inputs. An average accuracy of 84.2% was obtained in this research. Lee et al. (2019) applied a multimodal recurrent neural network approach to predict the transition from mild cognitive impairment to probable AD. The approach used an integrative framework that combines both the cross-sectional neuroimaging biomarkers at the baseline and the longitudinal cerebrospinal fluid/cognitive performance biomarkers obtained from the AD neuroimaging initiative cohort. When integrating longitudinal multi-domain data, the multi-modal deep learning method obtained the best accuracy of 81% and an AUC of 0.86 (Lee et al., 2019). Ding et al. (2019) developed a CNN based approach for early prediction of AD using PET brain images, showing an 82% specificity at 100% sensitivity and an average of 75.8 months prior to the clinical diagnosis. Recent researches have shown the feasibility of developing useful tools based on deep learning for early stage diagnosis of neurodegenerative diseases with biomarkers, such as glucose metabolism and amyloids.

Finally, we would like to mention about some recent advances in the application of machine learning on MD simulation and 3D structure modeling. Protein structure prediction tools such as Rosetta (Leaver-Fay et al., 2011) and Modeller (Webb and Sali, 2014), have been used in amyloid structure modeling. Skora and Zweckstetter (2012) predicted the amyloid fibril structure of the prion protein (PrP) fragment HET-s (218–289) with Rosetta and considered the chemical shift data as restraint inputs. Gu et al. (2016) determined a new Aβ42 protofilament model with Rosetta based on electron paramagnetic resonance data. Another study used Modeler to construct the dimeric structure of the human islet amyloid polypeptide (hIAPP) S20P mutant, an amyloid peptide related to type 2 diabetes, and performed REMD to study the dimerization pathway (Guivernau et al., 2016). Most traditional strategies of modeling and structure prediction are based on residue alignment of homologous protein or the so-called template-based modeling. The interactions between residues are considered as constraints of the energy functions of model refinement. With deep learning methods, the co-evolutionary or co-variation information of residue pairs is calibrated and converted into a restraint to develop a residual contact map for improving the energy terms. As a famous example, Alpha-fold has adapted the combination of homologous modeling and the contact map generated by DeepMind algorithm and achieved a remarkable milestone in terms of the critical assessment of protein structure prediction (CASP)13 (Alquraishi and Valencia, 2019).

Several protein structure prediction tools have led to the development of deep learning methods; for example, Rosetta was used to develop a deep residual network for predicting inter-residue orientations and its use resulted in a considerable improvement in the accuracy of structural prediction (Yang et al., 2020). The benefit of machine learning for structural prediction would also provide help in modeling amyloid oligomers. Recently, application of machine learning to enhanced sampling methods has became common (Yang et al., 2019). An approach named “targeted adversarial learning optimized sampling (TALOS)” was developed (Zhang et al., 2019b). This deep learning algorithm boosts the evaluation of partition function and enhances the sampling efficiency toward the target state. Ribeiro et al. (2018) developed a deep learning framework named variational Bayes for enhanced sampling (RAVE), which can perform highly accurate probability distribution along the reaction coordinate. Wehmeyer and Noé (2018) employed the training neural network to minimize regression error in DMD. Even the trajectory analyses in MD simulation are benefited from machine learning tools. For example, Degiacomi (2019) trained neural network based on MD trajectories to generate plausible conformations. In his work, these newly obtained structures proved to be reasonably accurate and the conformational space of the protein was detailed. The deep neural network could also be used in reducing the dimensionality of conformational spaces and improve the clustering representing the free energy landscape (Zhang et al., 2019a). All these observations imply that machine learning can be appropriately partnered with MD simulations. We believe that the combination of machine learning techniques and enhanced sampling methods will be used for studying the amyloid peptide in the near future, or such studies are even ongoing, and can provide new insights on the mechanism of oligomerization and fibrillization.

## Conclusion

The understanding of conformational dynamics of the Aβ peptides has been moving at a slow pace because of their transient character and intrinsic disorders. However, protein and peptide scientists globally have made enormous efforts to study the Aβ peptides. It is needed to develop and/or use various theoretical models and improve sampling techniques to explore the dynamics over a wide range of time scales. However, with the help of new experimental methods and efficient sampling methods using multiple force fields and representations, our knowledge about amyloid peptides in membranes, with and without ion metals, has significantly increased. In general, the results obtained from MD simulations of Aβ peptides depend strongly on the set of parameters used to describe the energy of the peptide and its interactions with the aqueous solvent. Further refinement and development of these important parameters, which describe the energy landscapes of Aβ peptides, would be required to uncover the mechanisms underlying many neurological disorders in the near future. In this review, we insist on development and testing of more sophisticated models to better understand the conformational dynamics of small acutely toxic Aβ monomeric peptides.

## Author Contributions

KS, HaZ, and HuZ are the co-first authors who drafted the manuscript. WX and YW are corresponding authors who supervised and revised the draft.

## Conflict of Interest

The authors declare that the research was conducted in the absence of any commercial or financial relationships that could be construed as a potential conflict of interest.
