# Supplementary material for: On the Conformational Dynamics of β-Amyloid Forming Peptides: A Computational Perspective
Source: Front Bioeng Biotechnol. 2020 Jun 3;8:532. doi: 10.3389/fbioe.2020.00532 (PMC7325929; doi:10.3389/fbioe.2020.00532)
Supplement: Supplementary file 1 [file Table_1.DOC]

Supplementary Table 1

**List of Neural disorders caused by β-amyloid extracted by text mining from Pubmed**

| AGNOSIA | DEAFNESS | LAFORA DISEASE | PARAPLEGIA |
| --- | --- | --- | --- |
| ALZHEIMER DISEASE | DELIRIUM | LETHARGY | PARKINSON DISEASE |
| AMNESIA | DEMENTIA | LISSENCEPHALY | POLIOMYELITIS |
| AMYOTROPHIC LATERAL SCLEROSIS | DERMATOMYOSITIS | MACHADO-JOSEPH DISEASE | POLYMYOSITIS |
| ANOMIA | DIABETIC NEUROPATHIES | MEMORY DISORDERS | POLYNEUROPATHIES |
| APHASIA | DOWN SYNDROME | MENINGITIS | PRADER-WILLI SYNDROME |
| ATAXIA | DYSKINESIAS | MENINGOENCEPHALITIS | PRION DISEASES |
| BLINDNESS | DYSTONIA | MENTAL RETARDATION | RETT SYNDROME |
| BRAIN EDEMA | ENCEPHALITIS | MOVEMENT DISORDERS | SCRAPIE |
| CADASIL | ENCEPHALOMALACIA | MULTIPLE SCLEROSIS | SEIZURES |
| CARPAL TUNNEL SYNDROME | ENCEPHALOMYELITIS | MULTIPLE SYSTEM ATROPHY | SLEEP DEPRIVATION |
| CENTRAL NERVOUS SYSTEM DISEASES | EPILEPSY | MUSCULAR ATROPHY | STATUS EPILEPTICUS |
| CEREBRAL AMYLOID ANGIOPATHY | FRAGILE X SYNDROME | MUSCULAR DYSTROPHIES | STROKE |
| CEREBRAL HEMORRHAGE | HALLUCINATIONS | MYOCLONUS | SUBACUTE SCLEROSING PANENCEPHALITIS |
| CEREBRAL INFARCTION | HEADACHE | MYOSITIS | SUBARACHNOID HEMORRHAGE |
| CEREBROVASCULAR DISORDERS | HEARING LOSS | NEURITIS | TAUOPATHIES |
| CHARCOT-MARIE-TOOTH DISEASE | HEMIANOPSIA | NEURODEGENERATIVE DISEASES | TOURETTE SYNDROME |
| CHOREA | HUNTINGTON DISEASE | NEUROFIBROMA | TUBEROUS SCLEROSIS |
| COMA | HYDROCEPHALUS | NEUROFIBROSARCOMA |  |
| CONFUSION | KURU | NEUROGENIC INFLAMMATION |  |
